# Linear Decrease in Athletic Performance During the Human Life Span

**DOI:** 10.3389/fphys.2018.01100

**Published:** 2018-08-21

**Authors:** Bergita Ganse, Urs Ganse, Julian Dahl, Hans Degens

**Affiliations:** ^1^Department of Orthopaedic Trauma Surgery, RWTH Aachen University Hospital, Aachen, Germany; ^2^Department of Physics, University of Helsinki, Helsinki, Finland; ^3^School of Healthcare Science, Manchester Metropolitan University, Manchester, United Kingdom; ^4^Institute of Sport Science and Innovations, Lithuanian Sports University, Kaunas, Lithuania

**Keywords:** athletics, performance, aging, throwing, jumping, sprinting, running, frailty

## Abstract

Master athletes maintain high physical activity levels and have better health than age-matched non-athletes. World records show accelerated declines after age 70 in swimming, long-distance running and sprint performance. However, less is known about age-related performance declines in the general master athlete population and whether decline rates differ between disciplines and genders. We interrogated a dataset including all track and field athletes of North Rhine from 2001 to 2014 to assess age-related changes in performance. 27,088 results of athletes between 11 and 89 years of age in 12 disciplines were analyzed by regression statistics. The analyses showed an accelerated decline beyond the age of 70 in sprint, middle- and long-distance running, while in throwing and jumping disciplines the performance continued a linear decline. Patterns of decline differed between men and women. The steepest declines were observed in javelin throw and 400 m (women), and in pole vault and 800 m (men). In conclusion, performance declines in aging depend more on the specific profile of requirements than previously assumed.

## Introduction

The “European Innovation Partnership on Active and Healthy Aging" predicts an increase in the number of people aged 65^+^ in the EU from 85 million in 2008 to 151 million in 2060^1^. An increased ratio of older people dependent on the healthcare system versus the working population contributing to the healthcare system is projected from 28% in 2014 to 50% in 2060 in the Western world (Booth and Hawley, 2015). While the lifespan has increased steadily, this is not the case for the healthspan (McPhee et al., 2016). Part of the problem of the disparity between increased lifespan and healthspan maybe the consequence of low physical activity levels (McPhee et al., 2016) and increased sedentary behavior in the older person (Wullems et al., 2016). Master athletes, on the other hand, maintain high levels of physical activity and retain a better health than age-matched non-athletes (Kettunen et al., 2006) and thus provide a model for the best attainable trajectory of aging (Lazarus and Harridge, 2017).

Track and field athletics is one of the oldest sports and includes running, sprinting, throwing and jumping events. Track and field athletics is popular, and people of all ages participate in competitions. Although world records give a rough understanding of the age-related declines in performance (Baker and Tang, 2010; Knechtle and Nikolaidis, 2017), they only reflect performance of the most exceptional individuals (Cheng et al., 2016). A cross-sectional analysis of the performance of master athletes irrespective of their ranking would reveal age-related changes in active individuals in general.

Boccia et al. (2017) published a longitudinal analysis of athletes up to the age of 35 to predict top-level careers in long and high jumpers from plots of individual trajectories of performance against age. Knechtle and Nikolaidis (2017) found in a cross-sectional study that ultramarathon performance peaks between 20 and 35 years of age and is followed by an accelerated decline after the age of 75 years. As each event has its specific requirements regarding speed, power, endurance, agility, coordination etcetera, the rates and patterns of decline may differ between different track and field events. Nevertheless, world records show accelerated declines after the age of 70 in as diverse disciplines as swimming and sprint (Berthelot et al., 2012; Korhonen et al., 2015), suggesting the age-related decline in performance is a general phenomenon, affecting all systems similarly. However, again, this is based on world records, and until now no studies systematically compare trajectories of the age-related decline in performance between disciplines. For a better understanding of the aging process, an analysis of aging-related trajectories of performance decline in several disciplines, irrespective of athletic standing, would be desirable. Patterns of decline would show the maximal compression of morbidity possible by exercise (Lazarus and Harridge, 2017), which maybe particularly important in women who have a lower muscle mass than men, and age-related muscle wasting may thus cause them to cross the disability threshold earlier (Degens and McPhee, 2013). It remains to be seen, however, whether differences in the age-related rates of decline in track and field athletics performance exist between male and female master athletes.

To address these questions, we interrogated a large data set including all registered athletes of North Rhine in Germany over many years to assess the trajectory of age-related changes in performance in several athletic disciplines. The hypotheses were: (1) the pattern of decline does not differ between events and (2) between men and women, (3) there are no differences in peak performance age between disciplines and genders, while (4) a more rapid decline in performance occurs after the age of 70. We also had the opportunity to analyze longitudinal changes in performance in several athletes who competed over many years and hypothesized no differences between longitudinal and cross-sectional changes in performance.

## Materials and Methods

Ethical approval was obtained from RWTH Aachen University Hospital IRB (reference number EK 300/17, date of approval: October 11, 2017).

### Generation of Data-Set and Data Availability

Athlete performance data was extracted from the official rankings lists of annual best results of each discipline in 2001–2014 of North Rhine Track and Field Association. The datasets analyzed for this study can be found in the result list repository of LV Nordrhein and are publicly available in html-format under the following URL^2^. North Rhine is a part of Germany and has a total population of approximately 17.5 million. Data was extracted from html-files and automatically reformatted into a table of individual athletes’ year-by-year (absolute age) best performance values for each discipline, using a script written in Perl (**Supplementary File**). Data was sorted for analysis using the same script and times were re-formatted into seconds.

### Statistical Analysis

Each year’s result lists comprise the best 20 results in each age group in each discipline. In master athletes, due to low participation, there are always fewer than 20 athletes in the lists, which means everyone who participated showed up in the ranking list. In non-master athletes, the age groups were: 11, 12, 13, 14, 15, 16/17, 18/19, and 20–29 years of age (men/women = the main class). In Germany, master classes already start at age 30, while internationally they begin at 35. Master classes continue in 5-year categories for both women and men (30–34, 35–39 etc.).

In the non-master athlete groups, athletes only show up when they exceed a performance threshold, which means only the best appear in the rankings. This explains the jump decrease in performance between 29 and 30 years of age, visible in many of the graphs. Due to this phenomenon, we decided to perform regression analyses for athletes younger than 20 and older than 29, and to ignore the performance data of people between 20 and 29 years of age in the regression analyses. Nevertheless, we do show the 20–29-year data in the graphs. Linear regression was found to deliver the highest *R*^2^, indicating the best match compared to exponential, logarithmic and polynomic regression. A second regression analysis was conducted to analyze a possible accelerated decline beyond age 70. Here, data was normalized to performance at age 30. Again, linear regression showed higher *R*^2^ than non-linear regression. Performance decline in percent per year was computed for athletes between 30 and 69 years and those 70 years and older. An accelerated decline after the age of 70 was assumed if the difference in the slope of the regression line was larger than 0.25 (25% difference). Likewise, differences in the slope of the regression line larger than 0.25 between disciplines or genders reflected a different age-related rate of decline between disciplines or genders, respectively. In addition, a third regression analysis was done for athletes under 30 years and athletes between 30 and 69 years to calculate peak performance age for each discipline and gender. The following formula was used (regression equation: Y = aX + b): peak performance = (*b* < 30–*b* > 30)/(*a* > 30–*a* < 30).

## Results

**FIGURE 1:**
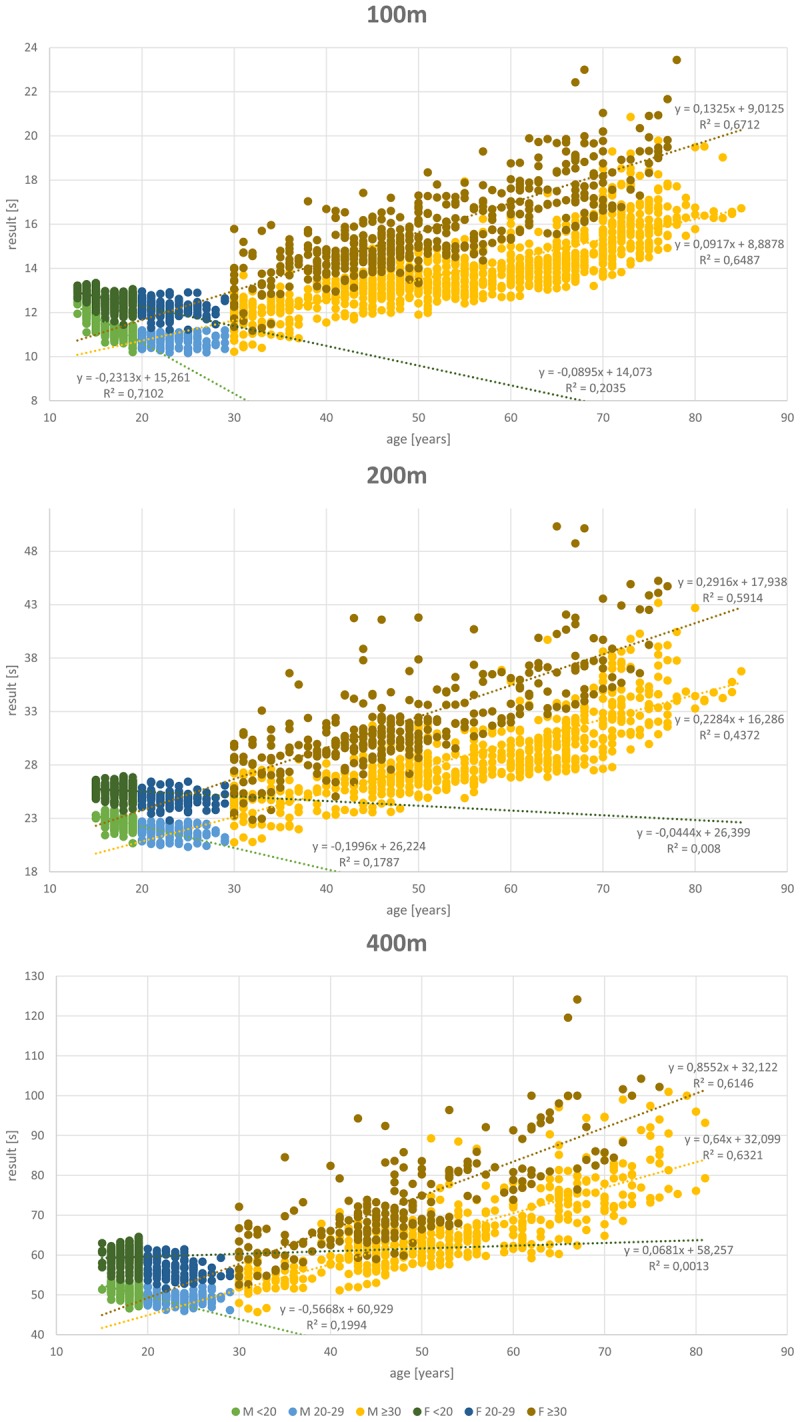
Regression analysis for the sprint disciplines (100, 200, and 400 m).

**FIGURE 2:**
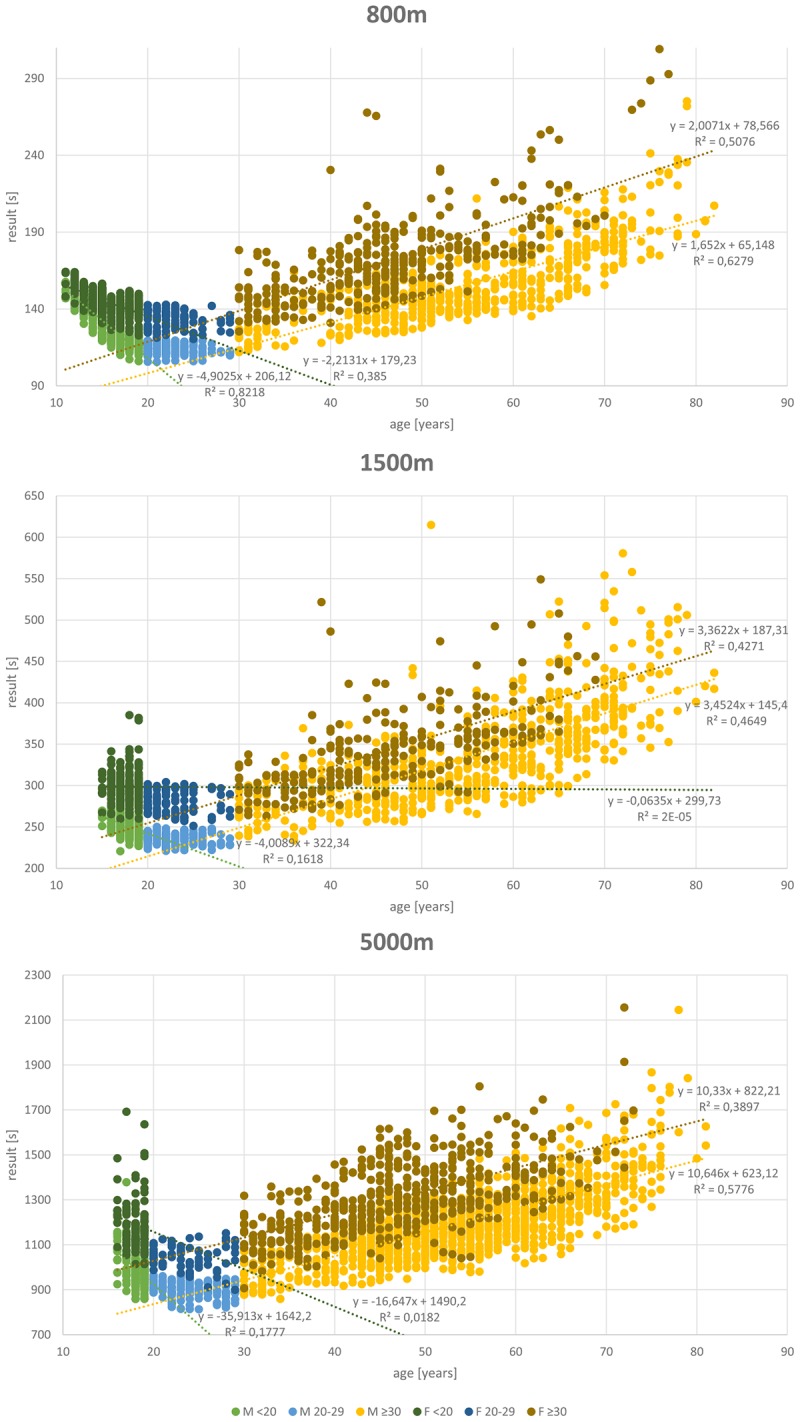
Regression analysis for middle and long-distance running (800, 1,500, and 5,000 m).

**FIGURE 3:**
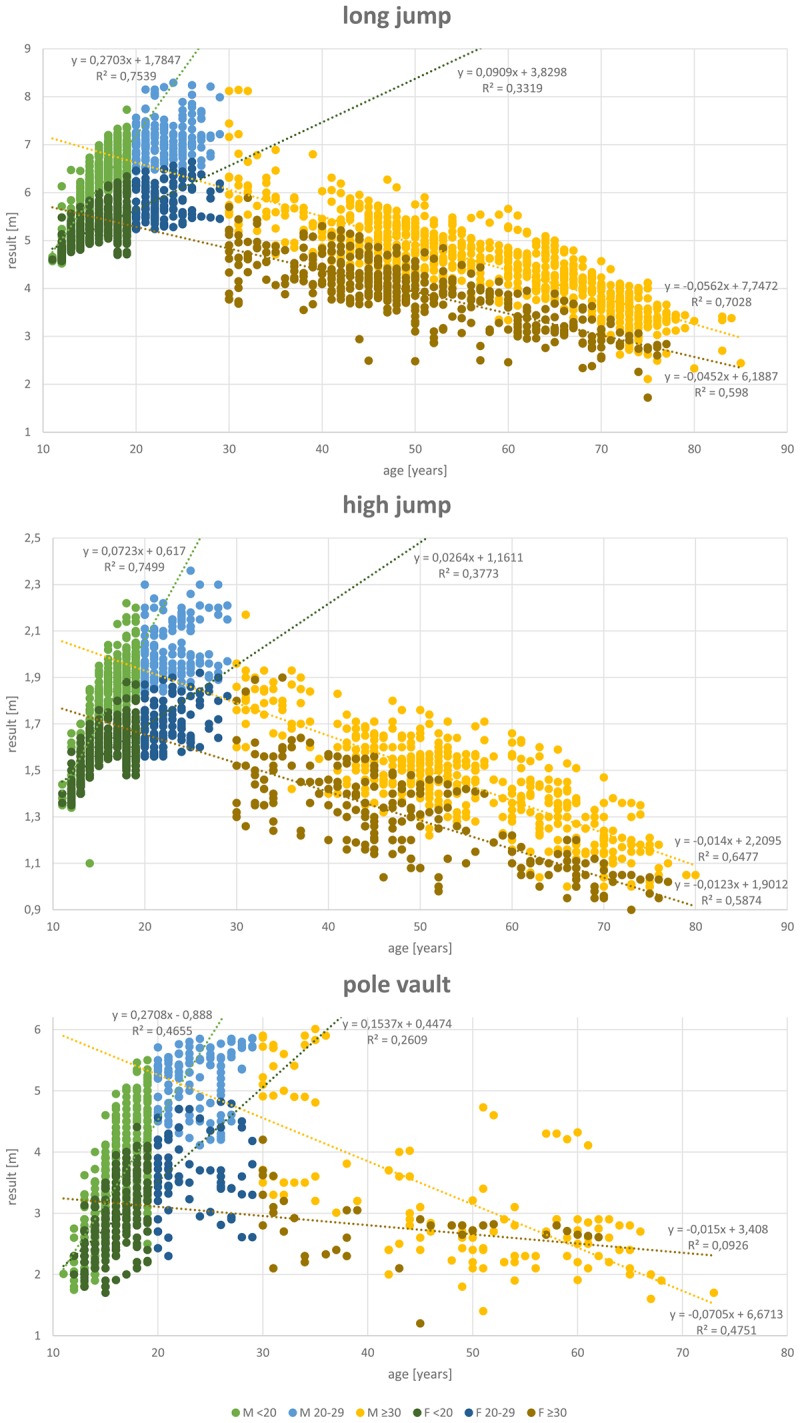
Regression analysis for the jumps (long jump, high jump, and pole vault).

**FIGURE 4:**
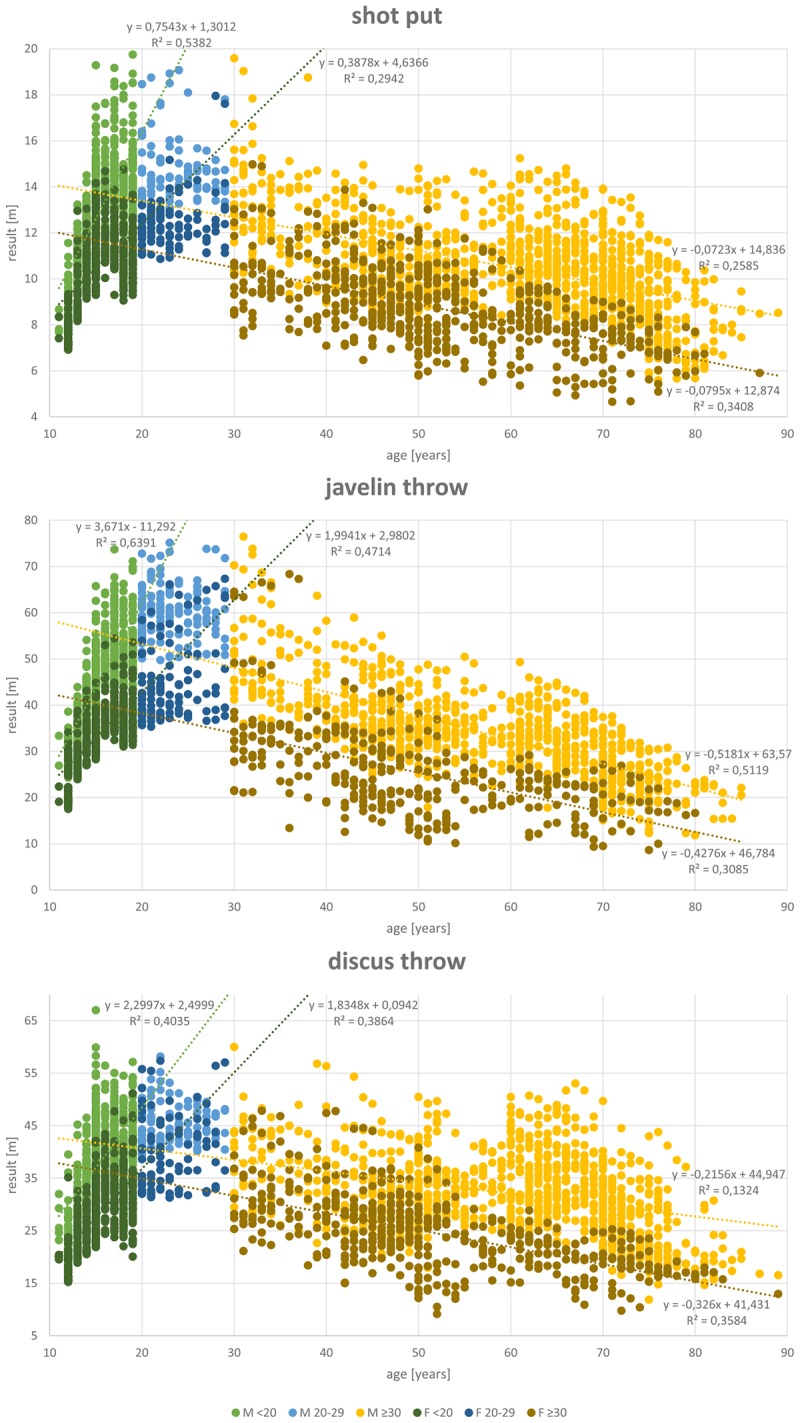
Regression analysis for the throws (shot put, javelin throw, and discus throw).

A total of 27,088 results of athletes between 11 and 89 years of age were included in the analysis. **Table 1** shows the data distribution over the different disciplines and between genders. Almost twice as many results are available from male (17,372) than female athletes (9,716). **Figures 1**–**4** display results of the regression analysis for sprints (**Figure 1**), middle and long-distance runs (**Figure 2**), jumps (**Figure 3**), and throws (**Figure 4**). The graphs show the regression equations for performance vs. age between 11 and 20, and 30–70-years, separated for men and women.

**Table 1:** Numbers of results in each event.

	F	M	Total
100 m	1,040	1,834	2,874
200 m	699	1,212	1,911
400 m	576	1,033	1,609
800 m	964	1,362	2,326
1,500 m	576	1,155	1,731
5,000 m	689	1,590	2,279
Shot put	1,113	1,875	2,988
Javelin throw	881	1,591	2,472
Discus throw	1,006	1,782	2,788
Long jump	1,004	1,651	2,655
High jump	821	1,386	2,207
Pole vault	347	901	1,248
Sum:	9,716	17,372	27,088

*F, female; M, male.*

### Participation in Different Disciplines

The most popular disciplines are 100 m, shot put, discus throw and long jump (**Table 1**). In the 400 m sprint (**Figure 1**), athlete numbers decreased after the age of 55 and in pole vault (**Figure 3**) very few athletes continue beyond age 30. **Figure 4** shows a transient reduction in participation in the throwing disciplines between 30 and 40 years of age, while an increase in participants occurs after the age of 60 in men but not in women. Disciplines with the oldest athletes appearing in the data set are shot put and discus throw (maximum age: 89 years).

### Age-Related Changes in Performance

**Figures 1**–**4** show a maturational increase in performance in all events followed by an age-related decline in performance. Results indicate the least variation in long jump where *R*^2^ is highest (men: 0.703, women: 0.598; **Figure 3**). In running disciplines, an accelerated decline beyond the age of 70 was found (**Table 2**). In the throwing and jumping disciplines, however, no such accelerated decline is present. The only exception was for the 200 m women, where the difference is not significant, probably because only 21 athletes were older than 69 years.

**Table 2:** Analysis for accelerated declines beyond the age of 70 and differences in the rate of decline in performance between 30 and 70-year-old male and female athletes.

Discipline	F < 70	F ≥ 70	Δ	% Δ	sign.	M < 70	M ≥ 70	Δ	% Δ	sign.	Δ_F,M_ < 70	% Δ_F,M_ < 70	Sign.
100 m	0.959	2.239	1.276	133	Yes	0.581	1.001	0.420	72	Yes	0.378	39	Yes
200 m	1.022	1.115	0.094	9	No	0.680	1.388	0.709	104	Yes	0.342	33	Yes
400 m	1.415	5.972	4.558	322	Yes	1.072	2.153	1.081	101	Yes	0.343	24	No
800 m	1.171	9.629	8.459	723	Yes	1.077	3.036	1.959	182	Yes	0.094	8	No
1,500 m	1.100	n/a	n/a			1.021	1.343	0.323	32	Yes	0.079	7	No
5,000 m	0.838	7.892	7.054	842	Yes	0.939	3.103	2.164	230	Yes	0.101	11	No
Shot put	0.788	0.693	−0.096	−12	No	0.363	1.503	1.140	314	Yes	0.425	54	Yes
Javelin throw	1.688	0.929	−0.759	−45	No	0.950	1.102	0.152	16	No	0.738	44	Yes
Discus throw	1.182	0.958	−0.224	−19	No	0.015	1.825	1.811	12,318	Yes	1.167	99	Yes
Long jump	0.944	1.144	0.2	21	No	0.752	0.857	0.104	14	No	0.192	20	No
High jump	0.843	0.256	−0.587	S-70	No	0.729	0.701	−0.028	−4	No	0.114	14	No
Pole vault	0.427	n/a	n/a			1.419	n/a	n/a			0.992	70	Yes

*Decline in percent per year normalized to the average result at age 30. Significance was assumed at 25%. A negative % shows a slower decline in performance after the age of 70. F, female; M, male; Δ, difference. %Δ, difference from initial decline; dark gray cells show a slowing of the age-related decline; %ΔF,M < 70, %difference from the gender with the highest slope; dark gray cells, men faster decline than women; light gray cells, women faster decline than men.*

### Differences in Age-Related Decrements in Performance Between Genders and Disciplines

**FIGURE 5:**
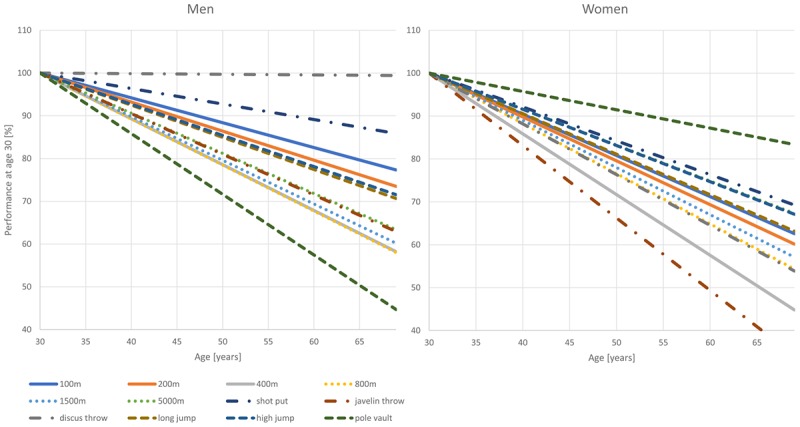
Comparison of regression lines of athletes 30–69 years in all disciplines and for both genders normalized to performance at 30 years (=100%).

**FIGURE 6:**
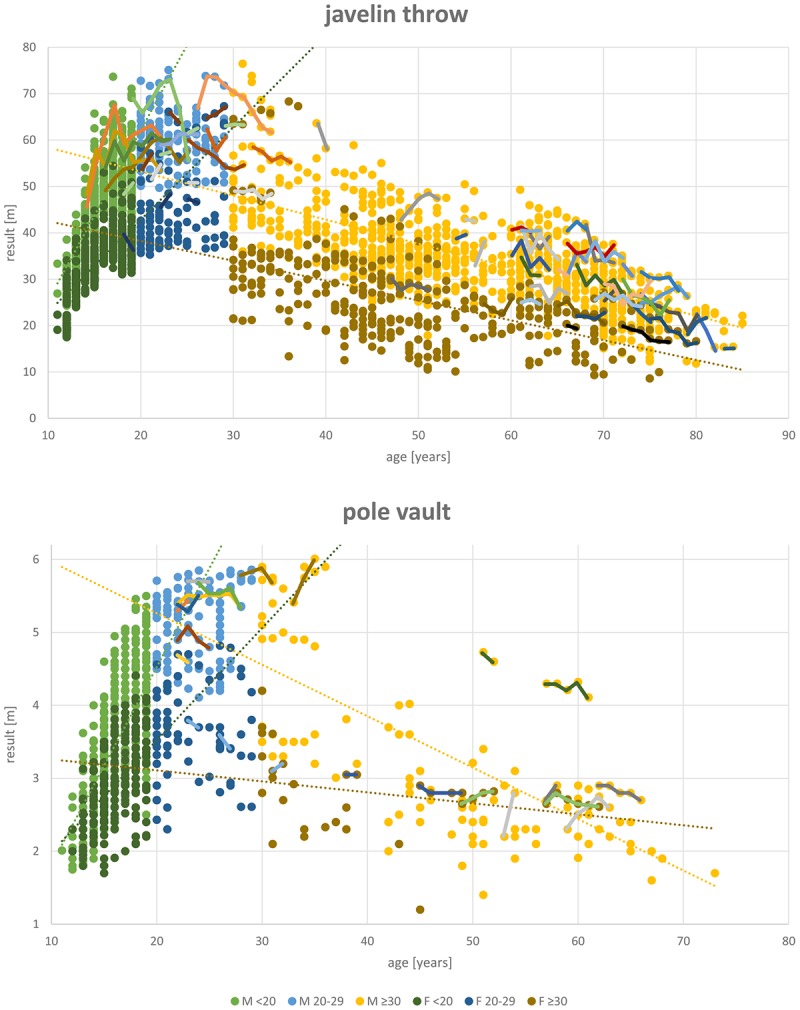
Overlay of the javelin throw and pole vault plots with curves of all athletes who have seven or more results in this data set.

The patterns of decline appear to differ between genders, as reflected by the differences in the annual percentage declines in performance in men and women (**Tables 2**, **3** and **Figure 5**). Performance shows the least steep decline in discus throw and 5,000 m running in women and in shot put and 100 m in men (**Table 2**). In men between 30 and 69 years of age, pole vault, 400, and 800 m show the steepest declines. In women, javelin throw, 400 and 800 m and discus throw decline fastest. We therefore analyzed javelin throw and pole vault further, applying an additional analysis to compare longitudinal and cross-sectional results. **Figure 6** shows longitudinal changes in javelin throwing performance of those individual athletes who appear at least with seven results in our data-set. Their declines follow the same pattern as observed in the cross-sectional data of the corresponding discipline in **Figure 4**. Student’s *t*-test revealed no significant difference between cross-sectional and longitudinal data (*p* = 0.254). While pole vault has the steepest decline of all events in men, it showed the slowest decline in women. **Figure 6** also shows a comparison of cross-sectional and longitudinal data for pole vault. All results in the women older than 45 years originate from the same athlete, indicating that the slower decline in pole vault performance in women needs to be interpreted with caution.

**Table 3:** Analysis of differences in rate of performance decline in percent per year (normalized to 30 years) between disciplines (upper number) and the delta/steepest slope of the pair (lower number) in 30- to 70-year-old athletes.

	100 m	200 m	400 m	800 m	1,500 m	5,000 m	Shot put	Javelin	Discus	Long jump	High jump	Pole vault
100 m		0.099	0.491	0.496	0.440	0.358	0.218	0.370	0.566	0.172	0.148	0.838
		14.5%	45.8%	46.1%	43.1%	38.1%	37.5%	38.9%	97.4%	22.8%	20.3%	59.1%
200 m	0.063		0.392%	0.397%	0.341	0.260	0.317	0.271	0.665	0.073	0.050	0.739
	6.2%		36.5%	36.9%	33.3%	27.7%	46.6%	28.5%	97.8%	9.7%	6.9%	52.1%
400 m	0.456	0.393		0.005	0.051	0.132	0.709	0.121	1.057	0.319	0.342	0.347
	32.2%	27.8%		0.5%	4.8%	12.3%	66.1%	11.3%	98.6%	29.8%	31.9%	24.5%
800 m	0.212	0.149	0.244		0.056	0.138	0.714	0.127	1.062	0.325	0.348	0.342
	18.1%	12.7%	17.2%		5.1%	12.8%	66.2%	11.8%	98.6%	30.2%	32.3%	24.1%
1,500 m	0.141	0.078	0.315	0.071		0.081	0.658	0.070	1.006	0.268	0.291	0.398
	12.8%	7.1%	22.2%	6.1%		7.9%	64.4%	6.9%	98.5%	26.2%	28.5%	28.0%
5,000 m	0.120	0.183	0.577	0.332	0.261		0.577	0.011	0.925	0.187	0.210	0.479
	10.9%	17.9%	40.8%	28.4%	23.7%		61.4%	1.2%	98.5%	19.9%	22.4%	33.8%
Shot put	0.171	0.234	0.627	0.383	0.312	0.050		0.588	0.348	0.390	0.367	1.056
	17.8%	22.8%	44.3%	32.7%	28.4%	6.0%		61.8%	95.8%	51.9%	50.3%	74.4%
Javelin	0.729	0.666	0.273	0.517	0.588	0.849	0.900		0.936	0.198	0.221	0.468
	43.2%	39.5%	16.1%	30.6%	34.8%	50.3%	53.3%		98.5%	20.8%	23.3%	33.0%
Discus	0.223	0.160	0.233	0.011	0.082	0.343	0.394	0.506		0.738	0.715	1.404
	18.9%	13.5%	16.5%	1.0%	6.9%	29.0%	33.3%	30.0%		98.1%	98.1%	98.9%
Long jump	0.014	0.077	0.470	0.226	0.155	0.106	0.156	0.743	0.237		0.023	0.666
	1.5%	7.5%	33.2%	19.2%	14.1%	11.2%	16.5%	44.0%	20.1%		3.1%	46.9%
High jump	0.115	0.178	0.571	0.327	0.256	0.005	0.055	0.844	0.338	0.101		0.689
	12.0%	17.4%	40.4%	27.9%	23.3%	0.6%	6.5%	50.0%	28.5%	10.7%		48.6%
Pole vault	n/a	n/a	n/a	n/a	n/a	n/a	n/a	n/a	n/a	n/a	n/a	

*Left/bottom, women; right/top, men. The % difference was calculated as the difference in slope between disciplines divided by the steepest slope. Dark gray indicates rate of decline in performance in parameter column larger than in the parameter in the row; light gray indicates decline in the performance of the parameter in the row declines faster than the performance of the parameter in the column.*

Results of the calculation of peak performance age are shown in **Table 4**. The discipline 400 m shows the highest age of peak performance in both genders. On average women peak approximately 2 years later than men (21.6 years vs. 19.7 years).

**Table 4:** Peak performance age calculated from regression equations.

	Peak F	Peak M
100 m	22.86	18.78
200 m	22.83	19.53
400 m	27.15	23.91
800 m	23.63	22.39
1,500 m	26.06	22.43
5,000 m	23.41	23.75
Shot put	18.61	15.75
Discus throw	19.70	13.06
Javelin throw	19.60	18.85
Long jump	17.21	18.57
High jump	19.92	19.54
Pole vault	18.23	23.09
Average	21.60	19.97

*F, female; M, male.*

## Discussion

In the present study, a dataset of athletics results was created from 14 annual ranking lists of North Rhine, Germany. In total, 27,088 results from athletes of all age groups in 12 disciplines were analyzed. The main findings are: (1) the age-related decline in performance accelerates after the age of 70 in the sprint and running disciplines, but not in throwing and jumping disciplines; (2) patterns of decline differ between events and (3) between men and women; (4) there are differences in peak performance age between disciplines and genders; (5) no significant differences were found between longitudinal and cross-sectional data.

### Patterns of Performance Decline

The performance in all disciplines showed a linear decline up to the age of 70 years. This is identical to the linear decline in V ^⋅^O_2_max (Tanaka and Seals, 2008) and power (Pearson et al., 2002) in master endurance and power lifter athletes that thus may underlie the age-related decline in performance we observed here. After the age of 70 years, however, the decline in the sprint and running but not in the throwing and jumping disciplines was accelerated, in accordance with results published previously (Tanaka and Seals, 2008; Rittweger et al., 2009; Baker and Tang, 2010; Lazarus and Harridge, 2017). In a review article on endurance exercise performance in master athletes, Tanaka and Seals (2008) describe progressively steeper declines at high age in marathon and swimming. Rittweger et al. (2009) analyzed master world records showing accelerated declines after age 70 in long distance, but less so in sprint disciplines. Baker and Tang (2010) compared performance declines in running, cycling, swimming, weightlifting, rowing, triathlon, walking and jumping, and assumed accelerated “curvilinear” declines with age for all these sports. We found an accelerated performance decline after age 69 in an analysis of a small group of male master javelin throwes (Ganse and Degens, in press).

In the throwing disciplines, such an accelerated decline in performance was not always seen. Part of the cause of the absence of an accelerated decline in performance in the throwing disciplines is that the implemented weights decrease over the years. In men, the last decrease of implement weights takes place at the age of 80 in shot put and javelin throw, and at 60 in discus throw. In women, at age 75 the last weight change occurs in shot put, discus and javelin throw. The implication for our analysis is that a similar distance thrown in older age in reality reflects a lower performance than a similar distance thrown at a younger age. Even so, also in the men’s shot put and discus throw there was an accelerated decline in performance after the age of 70, despite the decrease in the mass of the discus and put thrown. While the increase in numbers of athletes in these disciplines after retirement (Engberg et al., 2012; Schönbach et al., 2017) will result in an increased proportion of less-performing athletes that undoubtedly accentuate the population-based accelerated decline in performance after the age of 70, in the running disciplines participation numbers are not showing a large increase after the age of 70 (**Figures 1**, **2**). Thus, overall there appears to be an accelerated decline in performance after the age of 70 years. Lazarus and Harridge (2017) suggest that this pattern reflects a trajectory of a “fading integrative physiological capacity.” If the progressive age-related decline in bodily functions is a stochastic process, as seen in the accumulation of DNA damage (Cortopassi and Wang, 1996), then indeed it is to be expected that the decline in performance accelerates in old age (Degens, 2012).

As discussed above, the rate of decline was fastest in longer sprints and middle distances with relatively slow declines in throwing disciplines. Overall, our results indicate slower declines in anaerobic disciplines compared to aerobic disciplines. These results differ from those of Baker and Tang (2010) who compared performance declines in running, cycling, swimming, weightlifting, rowing, triathlon, walking and jumping, and found the fastest declines in weightlifting. Similarly, Gent and Norton (2013) found that in master cyclists in peak the anaerobic performance declined more than aerobic performance. Weightlifting is related to muscle power and should therefore closely compare to the throwing disciplines, especially shot put. However, in our data, shot put was the discipline with the least steep decline in men and an average rate of decline in women. A possible explanation for the different results might be the complexity of track and field disciplines that always require a mix of speed, agility, power and other factors. Another possible explanation might be the specific training track and field athletes undergo focussing on their disciplines, while Gent and Norton (2013) studied cyclists and tested them in ways that do not follow their usual training pattern. Baker and Tang (2010), however, studied results of the actual competition just like in our study.

Part of the discrepancy between the study by Baker and Tang (2010) and our study may be attributable to the different disciplines compared in theirs and our study suggesting part of the differences in the age-related rate of performance decline is attributable to factors other than changes in physiology, such as techniques, that modulate the age-related decline in performance. For instance, javelin throw shows the fastest decline of all throws and unlike other throwing events, not only arm, upper body and core strength are required, but also speed and agility (Kunz and Kaufmann, 1983). Pole vault, on the other hand, shows the fastest decline of all disciplines in men and is not only technically highly complex and demanding, but also requires the most diverse and extensive training (Linthorne and Weetman, 2012). While some studies have shown a faster decline in anaerobic power events and jumping than in aerobic and running events (Baker and Tang, 2010; Gent and Norton, 2013) we could not make such a clear distinction. It should be noted that Baker and Tang (2010) also found that some endurance events, such as cycling, and triathlon declined faster than the running and swimming performance. It does seem that not only strength or metabolism, but also changes in technique contribute to the age-related decline in performance.

### Gender Aspects

Gender differences in declines of track and field disciplines have to our knowledge not previously been reported. Although in our data set we had twice as many results of men compared to women and men competed to higher age, we still had a substantial set of data from female master athletes. Explanations for the lower participation of women are mainly cultural and relate to traditional role models and socio-economic status (Seabra et al., 2008; Toftegaard-Støckel et al., 2011). While male participation in the throwing disciplines increased after the age of 60, most likely due to retirement, this increase was not observed in women. Reasons could be the lack of an abrupt retirement wave of women in Germany, who work in general fewer hours and retire earlier than men (Leve et al., 2009). Another aspect for the lower participation of women is that while the first athletics competitions for women at Olympic Summer Games took place in 1928, most disciplines were only performed by women much later. The women’s long jump, 200 m and shot put showed up in 1948, 400 m in 1964, 5,000 m in 1996 and pole vault as late as 2000 (Schade et al., 2004). Nevertheless, there were no massive overall differences in the age-related decline in performance between genders, but rather varieties in detail. It is doubtful whether these differences in detail are real, as the lower numbers of female participants can probably explain most of gender-related differences in the rate of age-related decline in performance. Future studies are needed to confirm whether gender-related differences in the age-related decline in performance really exist.

### Peak Performance Age

The calculated peak performance age from regression data can only be rough estimates, especially because peak performance in elite athletes is to some degree determined by training intensities and support by clubs, family and sport organizations (Allen et al., 2015). In running, the age of peak performance has been reported to increase with race distance (Knechtle and Nikolaidis, 2018) and was estimated to be between 39 and 41 years in marathon and ultramarathon runners (Nikolaidis and Knechtle, 2018). In our analysis, we found much earlier peak performance in track and field disciplines, similar to the age of peak performance between 25 and 27 years for those disciplines observed before (Haugen et al., 2018). Haugen et al. (2018) observed a higher peak performance age in marathon runners and male throwers. Interestingly, similar to the observation by Haugen et al. (2018) we found that the age of peak performance was in general higher for women than men. However, if our calculations are correct, then the age of peak performance with regards to the disciplines analyzed in the present study (maximum 5,000 m) was highest for the 400 m sprint and not, as expected from the previous studies, in the longer distances. The discrepancy may be attributable to the fact that we included all athletes, while other studies only compared peak performers, and that we extrapolated the data. Whatever the cause of this discrepancy, the implication for coaches and officials is that talents in the long sprints and middle-distance disciplines need support for a longer period to reach their personal best than in other disciplines, such as in the jumps. High peak performance age also means collision with family planning and working life, and athletes in sports that peak late need more support in these areas if we want them to continuously perform well on international scale.

### Psychological Aspects

Apart from age-related decrements in physiological function, motivational changes across the athletic lifespan may also contribute to the age-related decline in performance (Medic et al., 2007). The authors showed that athletes are usually able to break records during the first 2 years in a 5-year age group and participation drops in the latter half of the 5-year age category, reflecting the influence of psychology at least on participation.

### Limitations

Our study is the first to analyze a large dataset regarding declines in performance of athletes in several athletics disciplines. The major strength of the study is the large amount of data. Though the data presented here is primarily of cross-sectional nature, the longitudinal data contained in the dataset follows the same pattern of decline as seen in the cross-sectional analysis. This indicates that cross-sectional analyses provide a good reflection of the age-related declines in athletic performance, and cross-sectional analyses may thus also give a good indication of age-related changes in other measures, such as muscle strength and maximal oxygen consumption. A potential weakness is that there were no athletes older than 89 in the dataset, precluding any firm conclusions on performance changes in the oldest-old master athletes.

## Conclusion

In conclusion, performance declines accelerated beyond the age of 70, particularly in runners and sprinters, while the real pattern of decline might be hidden in throwers due to decreasing implement weights. As age-related rates of decline differ between disciplines it indicates that the decline in performance is complex and dependent on both changes in physiology and technique. Significantly, the age-related decline seen in the few athletes whom we could follow longitudinally followed a similar time course compared to the cross-sectional data. The implication is that population wide cross-sectional studies give a good indication of the age-related changes in performance and physiology of athletes. Of course, individual factors such as comorbidities, genetics and lifestyle play major roles for individual development. Injuries and disease may stop athletes’ careers and decrease performance.

## Author Contributions

BG contributed the idea and worked on data analysis and interpretation, figures, tables, drafting, manuscript submission, and approval of the manuscript. UG worked on data collection, data interpretation, and approved the manuscript. JD worked on data collection and interpretation, and approved the manuscript. HD contributed to the statistical analysis, data discussion, drafting, and approval of the manuscript.

## Conflict of Interest Statement

The authors declare that the research was conducted in the absence of any commercial or financial relationships that could be construed as a potential conflict of interest.

## Footnotes

1 https://ec.europa.eu/eip/ageing/

2 http://archiv.lvnordrhein.de/index.php/wettkaempfe/bestenlisten/lvn-bestenlisten
