# Supplementary material for: Linear Decrease in Athletic Performance During the Human Life Span
Source: Front Physiol. 2018 Aug 21;9:1100. doi: 10.3389/fphys.2018.01100 (PMC6110907; doi:10.3389/fphys.2018.01100)
Supplement: Supplementary file 1 [file Table_1.DOCX]

The following code is a Perl-script to parse and format the official HTML result lists:

#!/usr/bin/perl -w

$class = "unknown"; # Age class

$disc = "unknown"; # Discipline

$format = 0; # 0 = html-ized, 1 = raw

# Iterate through all files

while(<>) {

$year = $ARGV;

$year =~ s/^20([0-9][0-9]).*/1$1/;

if(($year > 109 && $ARGV !~ /normal/) || $year > 111) {

$/ = "\n";

$format = 1;

} else {

$/ = "<pre>";

$format = 0;

}

chomp;

# Normalize umlauts and spaces

s/&ouml;/ö/g; s/&uuml;/ü/g; s/&auml;/ä/g;

s/&Ouml;/Ö/g; s/&Uuml;/Ü/g; s/&Auml;/Ö/g;

s/&szlig;/ß/g; s/&eacute;/e/g; s/&egrave;/e/g;

s/&nbsp;/ /g;

# Find new discipline tags.

if(/a name=\"?([^\">]*)/) {

#print STDERR " - - - Age group $1 - - -\n";

#$class = $1;

$p = $1;

if($p =~ /^(W|M)([0-9J][0-9AB])?([-0-9A-Z]{3}[M237K]?)$/) {

$gender = $1;

if(defined $2) {

$class = "$1$2";

} else {

$class = "$1";

}

$disc = "$3";

print STDERR "Found group $class discipline $disc.\n";

}

next;

}

# Throwing disciplines and high jumping

if($disc eq "SPE" || $disc eq "KUG" || $disc eq "HAM" || $disc eq "BAL" || $disc eq "DIS" || $disc eq "GEW" || $disc eq "STA" || $disc eq "HOC") {

# remove all tags, split on spans

$plain = $_;

$plain =~ s/<\/span>/#/g;

$plain =~ s/<[^>]*>//g;

if($format == 0) {$plain =~ s/\s+/ /g;}

if($plain =~ /^\s+$/) {

next;

}

if($format == 1) {

($distance, $name, $age, @values) = split(/\s\s+/,$plain);

} else {

($distance, $name, $age, @values) = split('#',$plain);

}

# Skip empty entries

if(!defined $name || $name =~ /^\s+$/) {

next;

}

# Make sure age was parsed correctly.

if(!defined($age) || substr($age,0,3) !~ /[0-9][0-9] /) {

next;

}

$age =~ s/([0-9]+) .*/$1/g;

$age = $year-$age; # <-- age is actually birthyear

if($age >= 100) {

$age -= 100;

}

$distance =~ s/,/./g;

$name =~ s/\s*$//;

$name =~ s/\s+/ /g;

$result{$disc}{$gender}{$name}{$age} = $distance;

$ages{$age} = 1;

print STDERR "Found entry: $name achieved $distance in discipline $disc at age $age\n"

}

# Jumping disciplines

if($disc eq "WEI" || $disc eq "DRE" ) {

# remove all tags, split on spans

$plain = $_;

$plain =~ s/<\/span>/#/g;

$plain =~ s/<[^>]*>//g;

if($format == 0) {$plain =~ s/\s+/ /g;}

if($plain =~ /^\s+$/) {

next;

}

if($format == 1) {

if(length($plain) < 14) {

next;

}

$distance = substr($plain,0,5);

($name, $age, @values) = split(/\s\s+/,substr($plain,13));

} else {

if(length($plain) < 14) {

next;

}

if(substr($plain,0,13) !~ /\(/) { # No wind

($distance, $name, $age, @values) = split('#',$plain);

} else {

$distance = substr($plain,0,5); # With wind.

($name, $age, @values) = split('#',substr($plain,13));

}

}

# Skip empty entries

if(!defined $name || $name =~ /^\s+$/) {

next;

}

# Make sure age was parsed correctly.

if(!defined($age) || substr($age,0,3) !~ /[0-9][0-9] /) {

next;

}

$age =~ s/([0-9]+) .*/$1/g;

$age = $year-$age; # <-- age is actually birthyear

if($age >= 100) {

$age -= 100;

}

$distance =~ s/,/./g;

$name =~ s/\s*$//;

$name =~ s/\s+/ /g;

$result{$disc}{$gender}{$name}{$age} = $distance;

$ages{$age} = 1;

print STDERR "Found entry: $name achieved $distance in discipline $disc at age $age\n"

}

# Running disciplines

if($disc =~ /[1-9](0|1|K)(0|5)H?$/ || $disc =~ /60H?/ ) {

# remove all tags, split on spans

$plain = $_;

$plain =~ s/<\/span>/#/g;

$plain =~ s/<[^>]*>//g;

if($format == 0) {$plain =~ s/\s+/ /g;}

if($plain =~ /^\s+$/) {

next;

}

if($format == 1) {

if(length($plain) < 14) {

next;

}

$time = substr($plain,0,7);

($name, $age, @values) = split(/\s\s+/,substr($plain,13));

} else {

if(length($plain) < 14) {

next;

}

if(substr($plain,0,13) !~ /\(/) { # No wind

($time, $name, $age, @values) = split('#',$plain);

} else {

$time = substr($plain,0,5); # With wind.

($name, $age, @values) = split('#',substr($plain,13));

}

}

# Skip empty entries

if(!defined $name || $name =~ /^\s+$/) {

next;

}

# Make sure age was parsed correctly.

if(!defined($age) || substr($age,0,3) !~ /[0-9][0-9] /) {

next;

}

$age =~ s/([0-9]+) .*/$1/g;

$age = $year-$age; # <-- age is actually birthyear

if($age >= 100) {

$age -= 100;

}

# Transform Time to seconds

if( $time =~ /([0-9]+:)?([0-9]+),([0-9]+)/) {

if( defined $1 ) {

$minutes = $1;

$minutes =~ s/://;

} else {

$minutes = 0;

}

$time = $minutes*60 + $2 + "0.$3";

} else {

print STDERR "Time in unknown format: '$time'\n";

}

$name =~ s/\s*$//;

$name =~ s/\s+/ /g;

$result{$disc}{$gender}{$name}{$age} = $time;

$ages{$age} = 1;

print STDERR "Found entry: $name achieved $time in discipline $disc at age $age\n"

}

}

# Determine minimum and maximum age in the results table

$minage = 9999;

$maxage = 1;

foreach $a (keys %ages) {

if($a > $maxage) {

$maxage = $a;

}

if($a < $minage) {

$minage = $a;

}

}

# Print result table

foreach $disc (keys %result) {

for $gender ("M","W") {

print "# - - - - Discipline $disc, $gender - - - -\n";

print "Name;\t";

for $i ($minage ... $maxage) {

print "$i;\t";

}

print"\n";

local %num;

local %sum;

local %ssum;

local %highest;

local %lowest;

for $i ($minage ... $maxage) {

$num{$i} = $sum{$i} = $ssum{$i} = $lowest{$i} = $highest{$i} = 0;

}

foreach $athlete (sort keys %{$result{$disc}{$gender}}) {

# Skip athletes that have less than 7 years of data

if(keys %{$result{$disc}{$gender}{$athlete}} < 7) {

next;

}

print "$athlete;\t";

for $i ($minage ... $maxage) {

if(defined $result{$disc}{$gender}{$athlete}{$i}) {

$val = $result{$disc}{$gender}{$athlete}{$i};

$num{$i}++;

$sum{$i} += $val;

$ssum{$i} += $val * $val;

if($val > $highest{$i}) {

$highest{$i} = $val;

}

if($val > $lowest{$i}) {

$lowest{$i} = $val;

}

print "$val;\t";

} else {

print " ;\t";

}

}

print "\n";

}

# Averages and variance

#print "[num];\t";

#for $i ($minage ... $maxage) {

# print $num{$i} . ";\t";

#}

#print "\n";

#print "[mean];\t";

#for $i ($minage ... $maxage) {

# if($num{$i} > 0) {

# print $sum{$i}/$num{$i} . ";\t";

# } else {

# print "0;\t";

# }

#}

#print "\n";

#print "[stddev];\t";

#for $i ($minage ... $maxage) {

# if($num{$i} > 0) {

# $mean = $sum{$i}/$num{$i};

# $stddev = sqrt($ssum{$i}/$num{$i} - $mean*$mean);

# print "$stddev;\t";

# } else {

# print "0;\t";

# }

#}

#print "\n";

#print "[max];\t";

#for $i ($minage ... $maxage) {

# print "$highest{$i};\t";

#}

#print "\n";

#print "[min];\t";

#for $i ($minage ... $maxage) {

# print "$lowest{$i};\t";

#}

#print "\n";

}

}

## Print simple two-column format

#foreach $disc (keys %result) {

# print "# - - - - Discipline $disc - - - -\n";

# print "# Age; # Result M; # Result W\n";

# for $i ($minage ... $maxage) {

# local @menresults;

# local @womenresults;

# foreach $athlete (sort keys %{$result{$disc}{"M"}}) {

# if(defined $result{$disc}{"M"}{$athlete}{$i}) {

# push @menresults,$result{$disc}{"M"}{$athlete}{$i}

# }

# }

# foreach $athlete (sort keys %{$result{$disc}{"W"}}) {

# if(defined $result{$disc}{"W"}{$athlete}{$i}) {

# push @womenresults,$result{$disc}{"W"}{$athlete}{$i}

# }

# }

#

# while($#menresults > 0 || $#womenresults > 0) {

# print "$i; " . pop(@menresults). "; " . pop(@womenresults) . "\n";

# }

# }

#}
